# Successful Treatment of Low-Flow Vascular Malformation in the Lip Using Intralesional Bleomycin: A Case Report

**DOI:** 10.7759/cureus.74119

**Published:** 2024-11-20

**Authors:** Anudeep Gopishetty, K. Amarnath, D. P. Uma Magesh, G. Saichand

**Affiliations:** 1 Department of Oral and Maxillofacial Surgery, Meghna Institute of Dental Sciences, Nizamabad, IND; 2 Department of Oral and Maxillofacial Surgery, Sri Venkateshwara Dental College, Chennai, IND

**Keywords:** intralesional bleomycin, low-flow lesion, oral surgery, sclerotherapy, vascular malformation

## Abstract

Vascular malformations (VMs) are congenital abnormalities of blood or lymphatic vessels, present at birth and growing proportionally with the individual. They are classified into types such as capillary, venous, lymphatic, and arteriovenous malformation (AVMs). Symptoms include discoloration, swelling, pain, or functional impairment, depending on the type and location. Diagnosis involves imaging like ultrasound, MRI, or angiography, and treatment options include sclerotherapy, laser therapy, embolization, or surgery for symptomatic cases. It is a class of vascular anomalies, known as VMs are benign lesions that arise from aberrant vascular tissue and can result from either lymphatic proliferation or angiovascular dysregulation. High-flow VMs, such as AVMs, involve direct connections between arteries and veins. AVMs can be congenital, presenting at birth. They are typically persistent and progressive, with the potential to cause significant blood loss, which can be life-threatening. Several treatment modalities are available, including surgical excision, electrocautery, LASER therapy, cryotherapy, radiotherapy, and the use of locally injected agents. Among these, sclerosing agents are particularly advantageous due to their affordability, availability, and high efficacy. Commonly used sclerosing agents include ethanol, sodium morrhuate, sodium tetradecyl sulfate (STS), boiling contrast media, and bleomycin. This case report describes a VM with low-flow characteristics located at the commissure of the lip, which was successfully treated with intralesional bleomycin injections. The aim of this case report is to document the successful management of a low-flow VM at the lip commissure using intralesional bleomycin injections. It highlights the efficacy and safety of this minimally invasive treatment in achieving favorable cosmetic and functional outcomes. The study also seeks to contribute to the growing evidence supporting sclerotherapy as a reliable alternative to surgical intervention.

## Introduction

Arteriovenous malformations (AVMs) are congenital abnormalities of blood or lymphatic vessels, present at birth and growing proportionally with age. They can be classified into types such as capillary, venous, lymphatic, and arteriovenous malformations (AVMs). Symptoms include discoloration, swelling, pain, or functional impairment, depending on the type and location [1]. It was once believed that defective vascular architecture was present at birth and that vascular abnormalities developed in utero when the patient was physiologically inactive. These benign lesions can originate from capillary, venous, lymphatic, or arteriovenous structures. They should not be confused with vascular tumors characterized by endothelial hyperplasia, such as hemangiomas [1,2]. The involved vessel subtypes include combination/complex malformations, veins (VMs), arteries (AVMs), lymphatics (lymphatic malformations (LMs)), and capillaries (capillary malformations (CMs)). Malformations are further categorized based on flow dynamics: slow-flow or high-flow lesions. Slow-flow malformations can affect capillaries, veins, or lymphatics [2] and these subtypes exhibit distinct flow characteristics, influencing both clinical management and treatment strategies. In contrast, high-flow malformations often involve arteries and veins. Slow-flow malformations may result in significant, lifelong complications, with clinical presentations varying according to the subtype and anatomical location affected. 

AVMs as a health condition have been reported to have a low burden in India with the approximated population prevalence of AVMs ranging between 0.02% and 0.05%, but (intracranial AVM is most likely reported). Studies carried out in hospital-based settings show that AVMs comprise 10%-15% of the vascular anomalies that are treated in some specialized centers found in infernal regions. The patients in this condition are diagnosed from 20 to 40 years with no gender dominance noted. Intracranial AVMs entail a risk of complications such as bleeding that can present in up to 50% of the patients at the time of presentation in which the patient may also experience a hemorrhagic stroke, seizures that occur in 30%-40% of the patients [2-4]. The progressive use of diagnostic imaging methods like CT angles or MRI angiography has also enhanced the rate of such detection. Still, access to comprehensive care services remains a challenge in the framework of rural development. Formulating strategies designed to improve early detection techniques as well as creating centers for cervical vascular anomaly treatment will be necessary for the management of the AVM burden in India [4,5].

Optimal treatment requires an individualized approach, guided by the lesion's type, size, location, and associated symptoms. Although surgical debulking was once the primary treatment, the development of minimally invasive techniques has expanded therapeutic options. Interventional procedures, such as sclerotherapy, embolization, and cryoablation, are now widely employed [3]. Sclerotherapy, in particular, involves injecting therapeutic agents directly into the affected vessels rather than relying on surgery, reducing risks and recovery time while minimizing damage to surrounding tissues. Several sclerosing agents have demonstrated efficacy, including boiling contrast media, bleomycin, sodium tetradecyl sulfate (STS), sodium morrhuate, and ethanol [4]. However, these agents can sometimes induce complications, such as severe tissue irritation, thrombosis, or localized inflammation. Intralesional bleomycin injection is a commonly employed therapy for benign cutaneous and subcutaneous lesions, including keloids and warts. Bleomycin, a well-known cytotoxic agent, was originally used systemically to treat various malignancies [5]. The primary mechanism of the drug involves oxidative DNA damage by binding to iron-containing metal ions and forming metallobleomycin complexes. These complexes generate reactive oxygen species, which induce both single- and double-strand DNA breaks and produce free-base propenals - particularly from thymine-via cleavage at the 3'-4' linkages [6]. A streptomyces verticillus-derived cytotoxic antibiotic is referred to as bleomycin. Owing to its anti-tumor and sclerosing properties, it has found extensive use in the treatment of several clinical conditions [3,4]. Though this drug was first known and used systemically for the treatment of various cancers, including but not limited to Hodgkin’s disease, testicular carcinoma, and squamous cell carcinoma, it has found a secondary use in the management of non-malignant conditions as well. In dermatology, for instance, intralesional bleomycin is administered for the treatment of warts and keloids because of its ability to cause oxidative DNA damage which ultimately results in cell death in the affected tissues [5]. Likewise, its utility in the treatment of lymphatic and, VMs has also grown, notably in-flowing low lesions. In spite of these VMs being surgically and/or embolization treated, intralesional bleomycin sclerotherapy has been effective and minimally invasive in further reducing the lesion and its associated symptoms without compromising the cosmetic and functional appearance [6]. With respect to AVMs, which are abnormal connections between arteries and veins that enable high blood flow, results have shown that bleomycin may also be effective. These malformations could be fatal; hence, surgery alone or together with embolization may not be the best treatment option for them, injections of bleomycin have been used as an effective adjunct treatment modality in those instances, where the surgical excision cannot be performed [5,6]. The use of bleomycin in the malformation aids to cause scarring that minimizes bleeding and enhances the vascular stability thereby allowing treatment of the lesions over the long term with minimal “side effects” [5-7]. The generation of free base propenals is known to trigger cell cycle arrest in the G2 phase, which inhibits cell replication and disrupts tissue development and repair. In recent years, intralesional bleomycin injections have also shown promise in the treatment of peripheral VMs, yielding favorable clinical outcomes [4,5].

The aim of this case report is to document the successful management of a low-flow VM at the lip commissure using intralesional bleomycin injections. It highlights the efficacy and safety of this minimally invasive treatment in achieving favorable cosmetic and functional outcomes. The study also seeks to contribute to the growing evidence supporting sclerotherapy as a reliable alternative to surgical intervention.

## Case presentation

**Figure 1:**
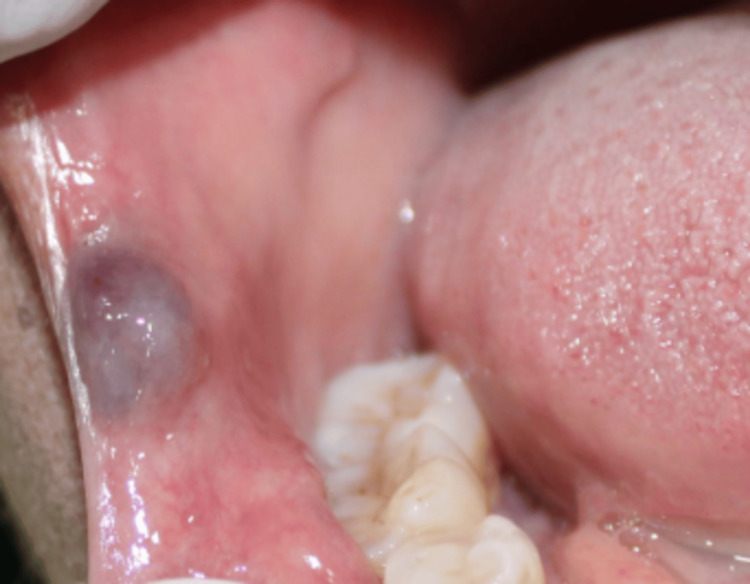
Intra-oral photograph (preoperative clinical photograph) Pre-operative intraoral view showing a well-defined, vascular malformation at the buccal surface of the right lip commissure. The lesion presents with a bluish hue, indicating underlying vascular involvement.

A 60-year-old woman presented to the maxillofacial and oral surgery department with swelling at the right commissure of her lip, which had persisted for two months. The swelling was reported to be a small, painless lump and gradually increased in size over time. Intraoral examination revealed a single, well-defined, sessile lesion with a bluish hue (Figure 1). It was located on the buccal surface of the right lip commissure and measured approximately 1.5 × 1.5 cm. Palpation confirmed the lesion was smooth, and pulsatile, with a variable consistency ranging from soft to firm, and non-reducible. On compression, the lesion demonstrated refill capacity, suggesting a vascular origin. Based on these findings, a provisional diagnosis of a VM was made.

**Figure 2:**
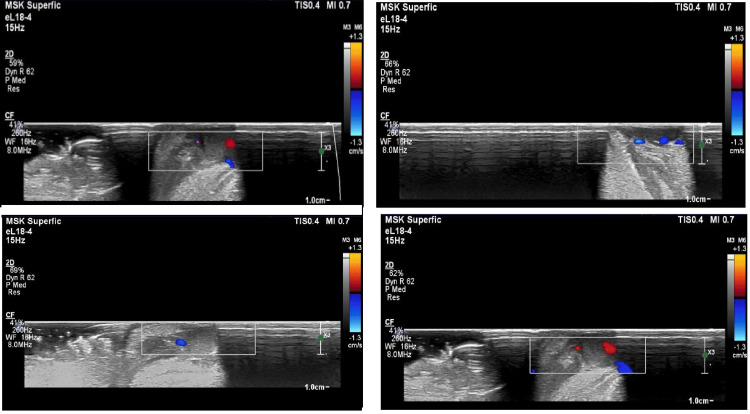
Ultrasound scan images of the case The squared boxes area shows the sluggish flow of blood

Doppler ultrasonography scan (Figure 2) confirmed an abnormality, indicating sluggish blood flow within the lesion. Considering the patient’s age and the size of the lesion, intralesional sclerotherapy with bleomycin under local anesthesia was selected to minimize surgical morbidity. Based on the observation in the ultrasound, the diagnosis of a low-flow VM located at the commissure of the lip was made. 

**Figure 3:**
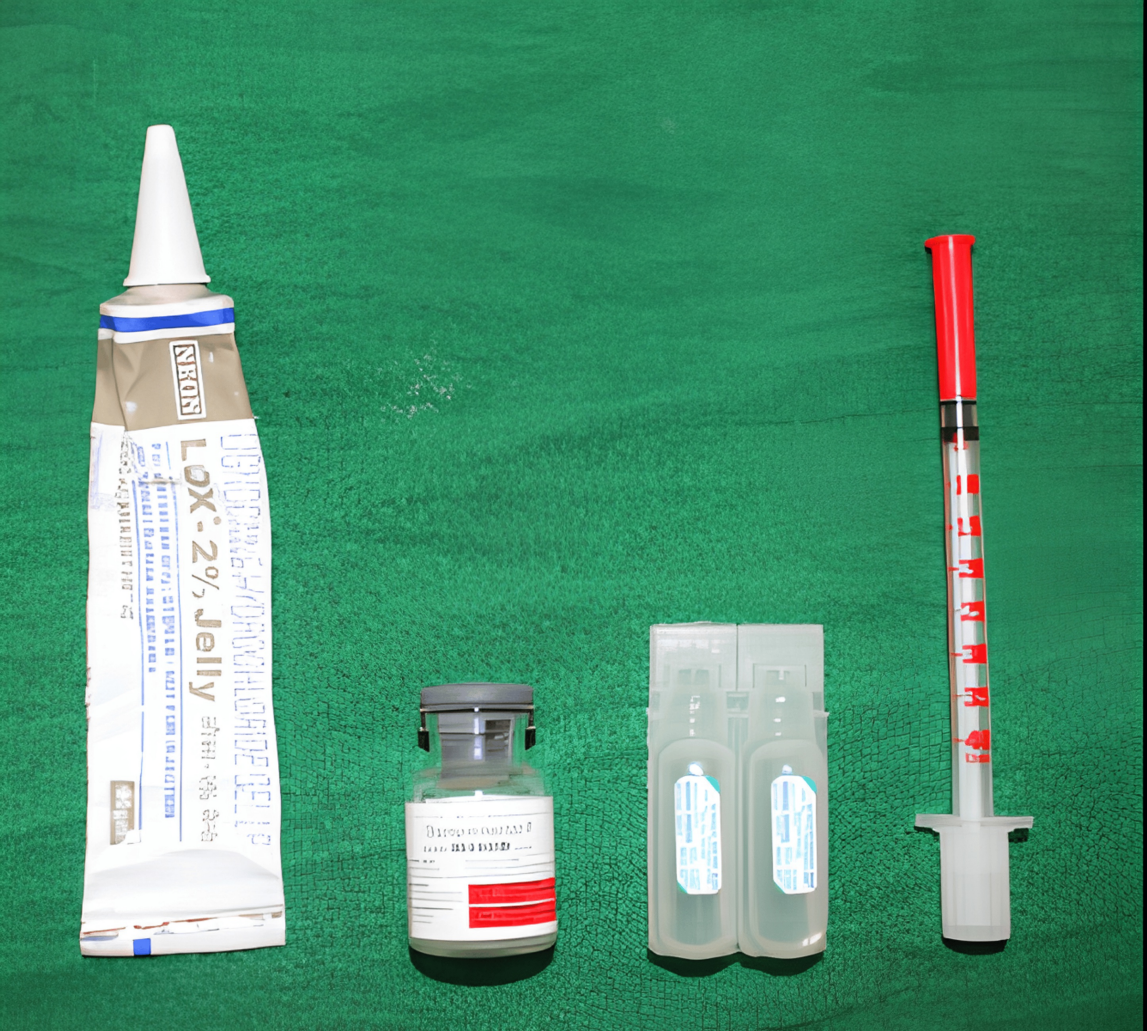
Armamentarium for local anesthesia and sclerotherapy It includes a 2% lignocaine jelly, bleomycin vial, sterile water for reconstitution, and a 25-gauge syringe for intralesional injection.

**Figure 4:**
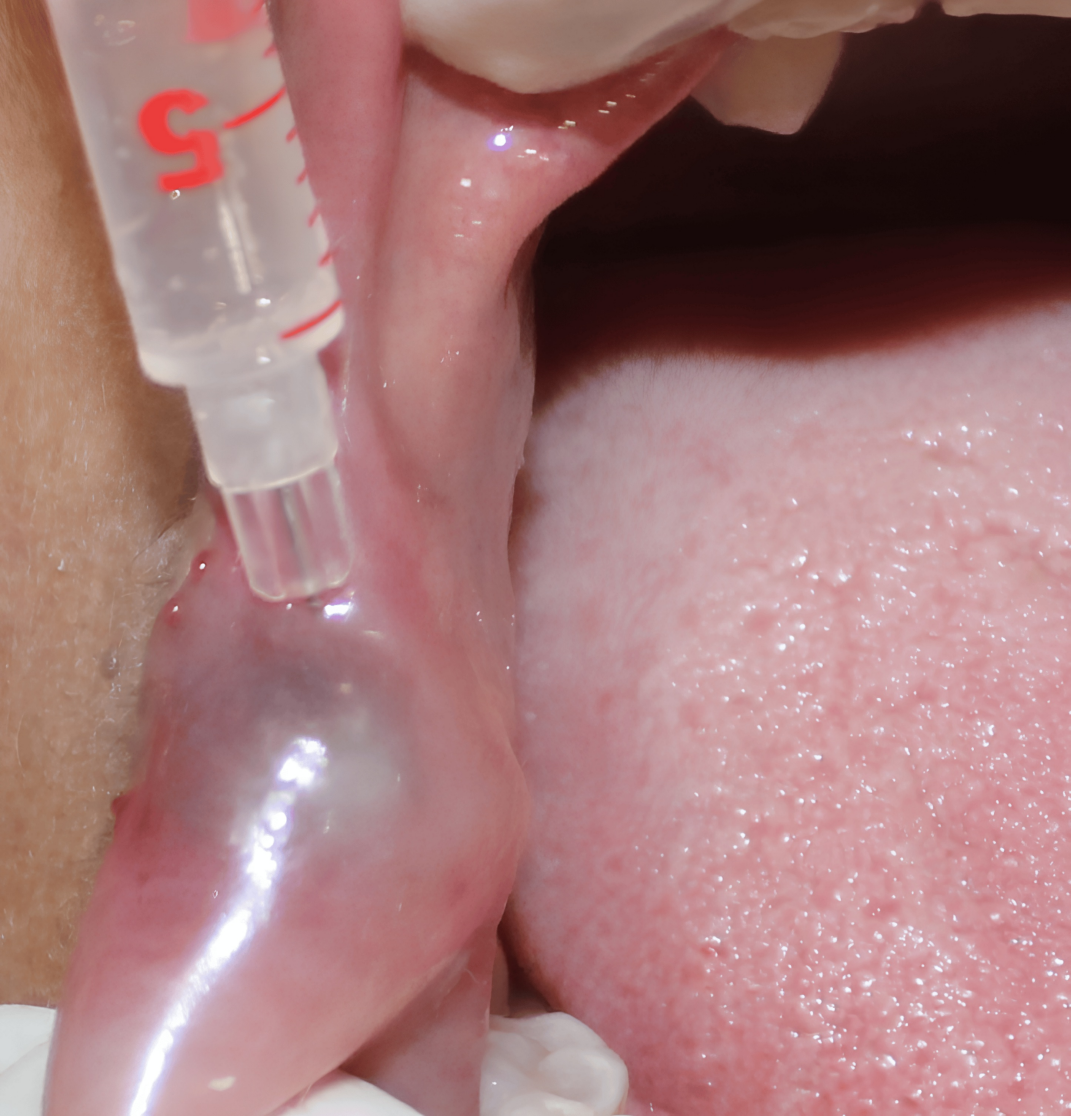
Intra-lesional injection of Bleomycin

The treatment plan consisted of two sessions. Prior to initiating treatment, the patient’s hematologic profile was assessed, with all parameters falling within normal ranges. In the first session, performed under aseptic conditions (Figure 3), local anesthesia was administered at the site. A 25-gauge needle was used to inject bleomycin intralesionally at multiple points in a circular pattern (dosage; 0.5 mg/kg, limit ≤ 15 mg/session). Bleomycin powder was reconstituted in 15 mL of distilled water, yielding a concentration of 1 mg/mL (Figure 4).

**Figure 5:**
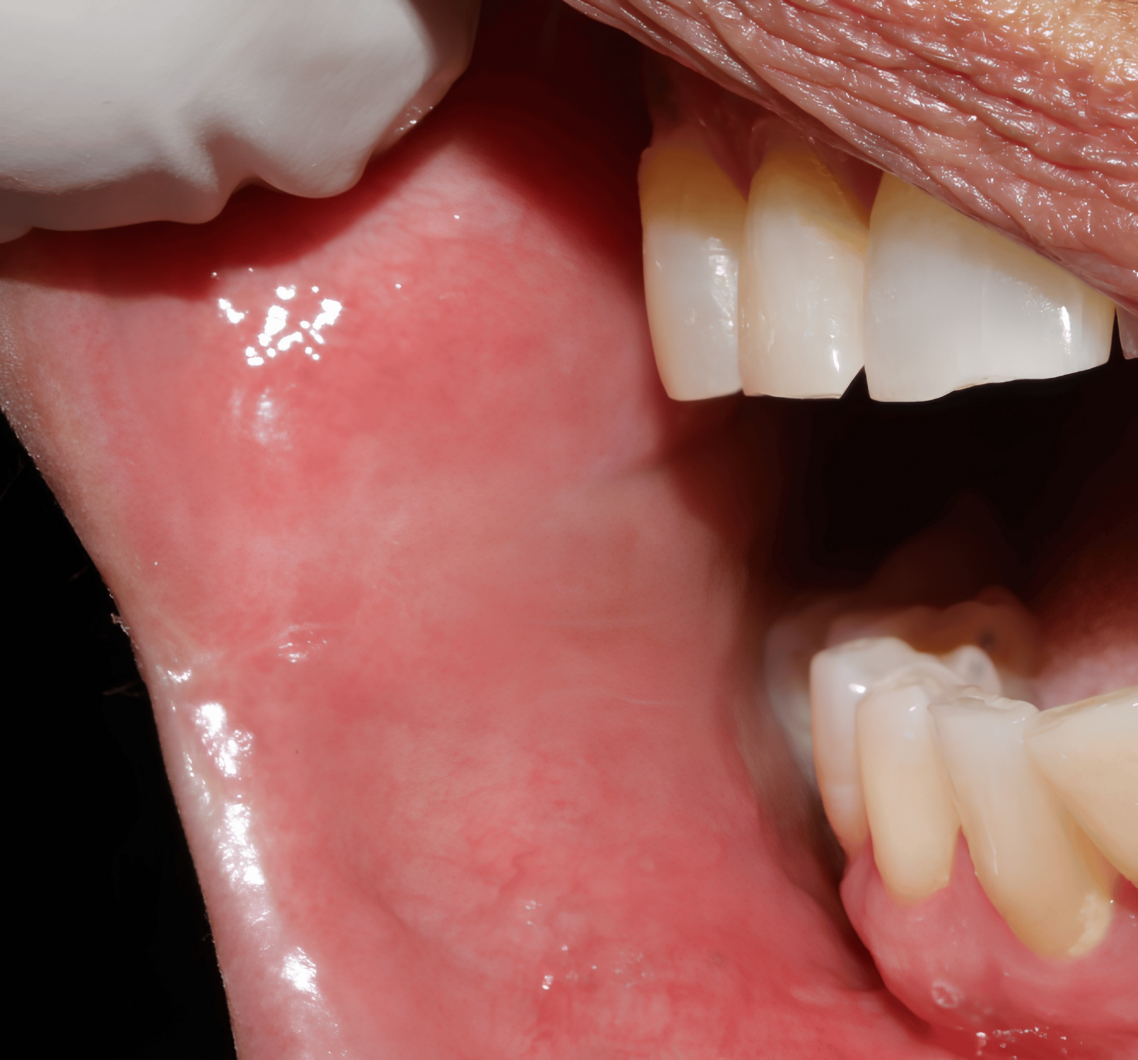
A complete post-operative recovery of the lesion captured after two months

The patient was prescribed a mouthwash containing chlorhexidine and placed on an antibiotic (Amoxicillin 500 mg x thrice a day x for five days) and an analgesic regimen (ibuprofen 200 mg x thrice a day x five days). A follow-up visit was scheduled two weeks later. At the follow-up appointment, the lesion was found to have decreased in size, and the patient reported no new complaints. The same injection procedure was repeated. After a third session, the lesion showed significant reduction, and by the fifth week, it had nearly resolved completely (Figure 5). The lesion healed without scarring or complications, and no further injections were required. The patient remained asymptomatic throughout the follow-up.

## Discussion

The management of VMs, particularly low-flow lesions, presents significant challenges and often requires a multidisciplinary approach involving radiologists, anesthetists, plastic and orthopedic surgeons, and vascular specialists. Head and neck regions are commonly affected by VMs [7], consistent with reports from previous studies. Additionally, conditions such as hemangiomas and AVMs are often observed in these areas, potentially contributing to their prevalence. Bleomycin has demonstrated efficacy in the treatment of low-flow VMs through intralesional injection. The concentration of bleomycin typically ranges between 0.5 and 1.5 mg/mL. It is widely used for systemic administration in diverse cancers such as Hodgkin’s disease, testicular cancer, and squamous cell carcinomas, among others. In non-cancers, intralesional bleomycin worked well on keloids, warts, and certain low-flow vascular pathologies as well as LMs. Its sclerosing action, which promotes fibrosis and regression of lesions, is its most beneficial aspect, especially in cases where surgical resection is not an option. The drug is typically used in adults and children; however, its dosing and administration will have to be modified with respect to age and weight in order to minimize toxicity [6-8]. Uses of bleomycin include advanced-age patients where the only limiting factor is a hostile tumor environment incorporating resection and prior surgery. Hypersensitivity reaction to the drug, lung impairment, or two pre-existing pulmonary diseases since it causes pulmonary fibrosis with a risk increasing to over 400 units cumulative dosage [7]. The elderly should also be cautioned since they are more prone to risk of pulmonary complications. Pro dosage is strictly based on body weight and careful monitoring to prevent adverse effects is observed. Most children can only receive a smaller total drug dose over the treatment period and intralesional bleomycin therapy shows good results in children. Patients with renal or hepatic impairment are less suitable for treatment as drug clearance is compromised hence underscoring the necessity of personalized treatment [8]. In adults, the recommended dosage is 10-15 mg per session, with a cumulative total not exceeding 100 mg. Previous studies have reported that cumulative doses in adults vary between 0.2 and 85 mg, while children may receive between 0.3 and 39 mg throughout treatment. Although sclerotherapy is generally considered a straightforward and effective treatment, caution must be exercised [9]. To avoid necrosis of surrounding tissues, the sclerosing agent should be injected precisely into the center of the lesion using a fine-gauge needle, such as an insulin syringe. The volume of each injection depends on the lesion’s size but should generally not exceed 2 mL [10]. Multiple sessions may be required, with follow-up assessments at one- to two-week intervals to determine the need for additional doses [11]. Table 1 shows the discussion of other published works.

**Table 1:** Review and discussion of several similar studies

Study (author)	Discussed and reviewed
Greene & Goss (2018)	Vascular malformations, particularly in the head and neck, are commonly reported and often require a multidisciplinary approach.
Carqueja et al. (2018)	Low-flow vascular malformations are challenging to manage and require tailored strategies, often involving sclerotherapy as a viable option.
Behravesh et al. (2016)	Discussed venous malformations and the role of interventions, such as sclerotherapy, in treatment plans.
Queiroz et al. (2014)	Reported successful sclerotherapy for haemangiomas, including oral cases.
Blum (1973)	Reviewed bleomycin as an antineoplastic agent and highlighted its use in systemic and intralesional treatments.
Petering et al. (1990)	Explained the mechanism of bleomycin’s action involving redox-active metals and its therapeutic impact.
Mulliken & Glowacki (1982)	Provided a classification system for haemangiomas and vascular malformations based on endothelial characteristics.
Sarihan (1997)	Demonstrated that intralesional bleomycin is effective in treating complex cutaneous haemangiomas, particularly in children.
Aitha et al. (2015)	Sclerotherapy, using precise administration of bleomycin, was shown to be a conservative and effective treatment for oral haemangiomas.
Burrows (2013)	Emphasised the use of endovascular techniques for the treatment of slow-flow vascular malformations, focusing on injection precision to minimise complications.
Trivedi et al. (2015)	Discussed the management of intraoral haemangiomas and the importance of multiple treatment sessions and follow-ups for effective outcomes.

## Conclusions

The management of VMs in the oral cavity remains complex, with inappropriate treatment often leading to poor outcomes. Sclerotherapy offers a minimally invasive and effective therapeutic option when appropriately indicated. In the present case, the use of bleomycin resulted in safe and effective lesion involution, highlighting the potential for non-surgical treatment to achieve favorable functional and cosmetic outcomes. This case illustrates the successful resolution of a VM using bleomycin sclerotherapy, underscoring its utility in achieving lesion regression without surgical intervention.
